# Integrated CRISPR-Cas12a and RAA one-pot visual strategy for the rapid identification of *Streptococcus equi* subspecies *equi*

**DOI:** 10.3389/fcimb.2025.1526516

**Published:** 2025-08-21

**Authors:** Haoyu Zu, Rongkuan Sun, Jiaxin Li, Xing Guo, Min Wang, Wei Guo, Xiaojun Wang

**Affiliations:** ^1^ State Key Laboratory for Animal Disease Control and Prevention, Harbin Veterinary Research Institute, Chinese Academy of Agricultural Sciences, Harbin, China; ^2^ College of Veterinary Medicine, Nanjing Agricultural University, Nanjing, China; ^3^ Institute of Western Agriculture, Chinese Academy of Agricultural Sciences, Changji, China

**Keywords:** strangles, *Streptococcus equi* subspecies *equi*, visual detection, RAA, CRISPR/Cas12a

## Abstract

Strangles, a highly contagious disease caused by *Streptococcus equi* subspecies *equi* (*S.equi*), significantly impacts horse populations worldwide, with Iceland as the only exception. This disease poses serious threats to equine health and results in considerable economic losses. Consequently, the accurate, sensitive, and rapid detection of *S.equi* from clinical samples is essential for early warning and effective disease management. This study introduces a novel detection method that integrates recombinase-aided amplification (RAA) with CRISPR/Cas12a technologies. We specifically designed RAA primers and CRISPR RNA to target the *eqbE* gene of *S.equi*, and we have carefully optimized the reaction systems for this purpose. The newly established visual diagnostic method has shown to be highly effective, demonstrating 97.14% specificity and 100% sensitivity, with the capability to detect as few as 5.6×10^0^ copies of the target. This is the first study to propose the combined application of RAA and CRISPR/Cas12a for the on-site rapid detection of *S.equi*. This is the first study to propose the combined application of RAA and CRISPR/Cas12a for the on-site rapid detection of *S.equi*, which enables visual point-of-care diagnosis of Strangles.

## 1 Introduction

Strangles, a prevalent infectious disease impacting horses worldwide, is attributed to *S.equi*. This disease results in economic losses exceeding £300,000 annually due to its widespread occurrence (Waller, 2014), and presents symptoms including fever, swelling, and rupture of the submandibular lymph nodes (Boyle, 2017; Dalgleish et al., 1993; Laus et al., 2007; Yelle, 1987). During outbreaks, even asymptomatic carriers can propagate the infection, primarily through nasal discharge, underscoring the necessity for meticulous monitoring. These carriers, while appearing healthy, can harbor and shed the *S.equi* pathogen for extended periods (Boyle et al., 2018; Riihimaki et al., 2018). Crucially, horses that have recovered can still transmit the infection for at least six weeks post-symptom resolution (Boyle et al., 2016), necessitating vigilant detection, isolation, and treatment to curb further spread.

The environmental persistence of *S.equi* varies; it survives only 1–3 days on surfaces like fences and soil but can remain viable in water for up to 4–6 weeks (Jorm, 1992; Mitchell et al., 2021; Weese et al., 2009). Transmission among horses can occur directly, through head-to-head or nose-to-nose contact, or indirectly via shared contaminated environments such as living quarters, water sources, and feeding equipment. Additionally, transmission can occur through contaminated tack, tethers, and even via the clothing and equipment of handlers and veterinarians, highlighting the need for comprehensive management strategies to control this infectious disease (Ladlow et al., 2006). As of 2019, six genetically distinct clusters of *S.equi* strains (BAPS-1 to BAPS-6) have been identified, with a global spread implicated in outbreaks across 19 countries involving 670 isolates. This widespread distribution underscores the critical need for the rapid and accurate diagnosis of *S.equi* to prevent Strangles outbreaks and control pathogen spread (Mitchell et al., 2021).

Although numerous diagnostic techniques for strangles exist, they still face limitations that affect their widespread application. The traditional method for isolating and identifying *S.equi* involves culturing samples on Columbia agar plates supplemented with 5% sheep or horse blood, with diagnoses based on the morphology of the hemolytic zone (Bannister et al., 1985; George et al., 1983; Woolcock, 1975). However, this method can lead to misdiagnoses due to other β-hemolytic *Streptococci*, such as *S. zooepidemicus*, which exhibit similar colony morphologies (Clark et al., 2008). Additionally, it is time-consuming and has relatively low sensitivity, increasing the risk of missed or incorrect diagnoses, and thereby affecting the effectiveness and timeliness of treatment (Boyle et al., 2018; Newton et al., 1997; Rendle et al., 2021). Diagnostic kits that utilize the SeM protein for indirect enzyme-linked immunosorbent assay (iELISA), such as those sold by Idvet, although useful, have limitations in effectively detecting horses in the recovery or pre-symptomatic phases, and fail to identify carriers (Boyle et al., 2009; Sheoran et al., 1997). Real-time quantitative PCR and PCR, while precise, are complex to operate and requires expensive instruments and equipment, limiting its use in field conditions, remote areas, or regions with scarce resources (Riihimaki et al., 2018; Newton et al., 2000; Baverud et al., 2007; Ijaz et al., 2012; Webb et al., 2013; North et al., 2014; Cordoni et al., 2015; Noll et al., 2020; Davidson et al., 2008; McLinden et al., 2023). Haoyu Zu’s study previously developed a real-time RAA method that can detect *S.equi* using portable instruments (Zu et al., 2024). Despite this advancement, there is still a need for a completely instrument-free method that is more suitable for on-site testing for the diagnosis of Strangles.

In recent years, recombinase-based isothermal amplification has emerged as a prominent alternative method for the detection of various pathogens, largely because of its simplicity and versatility, which facilitate applications in both field and laboratory environments. The mechanisms underlying recombinase polymerase amplification (RPA) and recombinase aided amplification (RAA) are consistent, encompassing binding, strand displacement, and extension processes to achieve DNA amplification *in vitro*. Ongoing technological advancements have augmented their benefits, including rapid execution within a temperature range of 37°C to 42°C, straightfward primer design, accelerated amplification kinetics, high sensitivity, minimal equipment requirements, operational simplicity, and the visual presentation of results. The evolution of these technologies has been critical for the development of innovative diagnostic methods for infectious diseases (He et al., 2022; Wang et al., 2022). RAA, in particular, has been successfully employed to detect adenoviruses, Akabane virus, enteroviruses, and respiratory syncytial virus, with these applications substantiated in the scientific literature (Ceruti et al., 2024; Ghosh et al., 2022; Kim et al., 2021; Zhang et al., 2023; Ma et al., 2021). Since its initial discovery in archaea in 1993, the CRISPR-Cas system has been identified in an expanding array of bacterial and archaeal genomes. Notably, CRISPR-Cas12 has garnered significant interest due to its potential for signal amplification and high target recognition capabilities in nucleic acid detection. Specifically, Cas12a can recognize specific nucleic acid targets under the guidance of RNA, activating its cis-cleavage activity for targeted cutting and its trans-cleavage activity, which indiscriminately cleaves all proximal single-stranded DNA. By integrating specific sequence single-stranded nucleic acid probes into the CRISPR-Cas system, the resultant cleavage effect amplification gives this technology the potential to serve as a groundbreaking nucleic acid diagnostic tool (He et al., 2020). It offers broad application prospects and has matured significantly, particularly when combined with LAMP, RPA, and RAA technologies. This integration not only enhances convenience but also significantly reduces the likelihood of false positives. Given its extensive applications in diagnosing infectious diseases, ensuring food safety, and conducting agricultural tests, this technology has attracted considerable interest from researchers (Heo et al., 2022; Huang et al., 2022; Jirawannaporn et al., 2022; Kellner et al., 2019).

In this study, we developed an integrated fluorescence visualization detection system based on recombinase-aided amplification (RAA) and CRISPR/Cas12a technologies. This innovative system requires only ultraviolet light for visualization, enabling the direct observation of diagnostic results for equine Strangles. This study is the first to combine RAA and CRISPR/Cas12a for the detection of *S.equi*. The method is not only cost-effective and easy to use but also demonstrates high sensitivity and specificity. Importantly, it also minimizes reliance on complex laboratory equipment. This diagnostic approach offers substantial practical benefits for the rapid identification of *S.equi*, crucial for preventing outbreaks of equine Strangles and reducing the related economic impacts.

## 2 Materials and methods

### 2.1 Pathogen strains, samples, and nucleic acid extraction

#### 2.1.1 Pathogen strains

The pathogens used in the experiments included equine influenza virus (H3 subtype), equine anemia virus, equine herpesvirus types 1 and 4, equine arteritis virus, and Escherichia coli. The *S.equi* strain (HJL2018) and *S. zooepidemicus* were acquired from the Harbin Veterinary Research Institute of the Chinese Academy of Agricultural Sciences. All strains underwent verification for purity and viability within a biosafety laboratory setting.

#### 2.1.2 Sample collection

The samples comprised nasopharyngeal swabs collected from horses suspected of suffering from Strangles. These clinical samples were provided by farm owners in collaboration with the Harbin Veterinary Research Institute of the Chinese Academy of Agricultural Sciences. Upon collection, the samples were immediately placed into sterile tubes containing a preservative solution and transported to the laboratory under refrigerated conditions.

#### 2.1.3 Nucleic acid extraction

Genomic DNA or RNA was extracted from the preservative fluid of the collected swabs using the TIANamp Bacteria Genomic DNA Extraction Kit Ver.3.0 (TianGen Biotech Co., Ltd., Beijing, China), in accordance with the manufacturer’s protocol. The extracted DNA quality was verified by NanoDrop spectrophotometry, showing A260/280 ratios of 1.8-2.0.

#### 2.1.4 Preparation and quantification of recombinant plasmid

Standard plasmid templates targeting the *eqbE* gene were engineered using the TSINGKE TSV-007VS pClone007 Versatile Simple Vector Kit (Beijing Tsingke Biotech Co., Ltd. Beijing, China). Post-construction the plasmids were purified using TIANprep Mini Plasmid Kit (TianGen Biotech Co., Ltd., Beijing, China). Subsequently, the purified plasmids were sequenced for verification (Saiwen Biotechnology Co., Ltd., Harbin, China). The purity and concentration of the plasmids were determined using NanoDrop OneC Spectrophotometer.

The copy number of the recombinant plasmids was calculated using the following formula:


$$C\;=\left[\left\{X*6.022\;*10^{23}\right\}/\{Y\;*\;660\;Da\}\right]$$

Where, “C”represents the copy number of the plasmid (copies/μL), “X” is the concentration of the plasmid (g/μL), “Y” is the number of base pairs in the target fragment, “660”is the average molecular weight of a DNA base pair in Daltons (Da), and 6.022×10^23^ is Avogadro’s constant.

### 2.2 Design and selection of RAA primers

#### 2.2.1 Selection of specific gene fragment

To ensure the specificity of the RAA reaction, the *eqbE* gene of *S.equi* was selected as the target amplification fragment. This gene is unique to *S.equi*. and does not share homology with other subspecies of *Streptococcus* such as *S. zooepidemicus*, guaranteeing the specificity of the amplification process.

#### 2.2.2 Primer design and synthesis

The *eqbE* gene sequences from 14 *S.equi* strains available in the NCBI database were aligned using the Geneious 9.0.2 (https://www.geneious.com) software. Based on this alignment, three forward and three reverse primers were designed using Snapgene 6.02 software. These primers were combined to form nine different pairs, and were synthesized by the Saiwen Biotechnology Co., Ltd. (Harbin, China). Details regarding the primer sequences and target lengths are provided in **Supplementary Table 1**.

#### 2.2.3 Selection process for primer pairs

To determine the most sensitive pair of primers, the recombinant plasmid containing 5.6×10^4^ copies of the target was used as the template for the RAA reaction. After the reaction, phenol-chloroform was used for DNA extraction. The extracted DNA was subsequently analyzed through 2% agarose gel electrophoresis to evaluate the clarity and positioning of the bands.

#### 2.2.4 RAA reaction conditions

The RAA reaction was performed using the RAA Rapid Amplification Kit (Qitian Biotech Co., Ltd., Suzhou, China) following the manufacturer’s instructions. The conditions were maintained at 37°C for 30 minutes. These design and selection processes ensured the specificity and efficacy of the primers, facilitating a successful RAA reaction and the generation of ample target DNA.

### 2.3 Design of crRNA

CrRNA contains a sequence that can specifically recognize target DNA, and a neck loop structure sequence which enables it to recognize and bind to Cas12a protein, thereby activating the cutting function of Cas12a. Therefore, crRNA plays a crucial role in the CRISPR/Cas12a cutting reaction. To enhance the sensitivity and specificity of the reaction, we designed five specific crRNAs (see **Table 1**), which were synthesized by Saiwen Biotechnology Co., Ltd.(Harbin,China), and used for subsequent screening. The design of these crRNAs was carried out using the online tool Benchling (CRISPR Guide RNA Design Tool | Benchling), which provides a comprehensive set of CRISPR design and analysis tools. This approach enabled the identification and cleavage of various target DNA sequences, allowing for the selection of the most optimized molecular tools through the screening of multiple candidates, and ultimately enhancing the sensitivity and specificity of the entire CRISPR/Cas12a system.

**Table 1:** Sequences of crRNAs generated and tested in this study.

Name	Sequence (5’- 3’)
*eqbE*-crRNA 1	UAAUUUCUACUAAGUGUAGAU*TTCGTTAATTCTTCG TCACCATG
*eqbE*-crRNA 2	UAAUUUCUACUAAGUGUAGAU*GCAACACCAACTCCA CCGATAAG
*eqbE*-crRNA 3	UAAUUUCUACUAAGUGUAGAU*AACATAATTAGGGC AGATCCTACC
*eqbE*-crRNA 4	UAAUUUCUACUAAGUGUAGAU*CCATTCCATATGG TAGACCTTTGA
*eqbE*-crRNA 5	UAAUUUCUACUAAGUGUAGAU*GAAAAATCAAGTG TATAGAGTTGT

*UAAUUUCUACUAAGUGUAGAU is the structural sequence of crRNA recognized by LbaCas12a.

### 2.4 Optimization and establishment of the CRISPR/Cas-eqbE-RAA platform

To enhance the enzymatic activity and visualization of the CRISPR/Cas12a system, we firstly tested all designed crRNAs to verify the feasibility of the reaction and confirm the contribution of each component, with the reaction system containing 50 nM Cas12a protein, 50 nM crRNA (pooled mixture), 120 nM reporter, 1× reaction buffer, and 20 ng template DNA. Fluorescence signal intensity was continuously monitored for 40 minutes using MA-688 Real-Time Quantitative Thermal Cycler under 470 nm ±10 nm (Suzhou Molarray Co., Ltd.), with recordings taken at one-minute intervals. The selection criteria included the peak fluorescence intensity (under 470 nm ±10 nm) and the clarity of the signal observable with the naked eye (under 365 nm UV light),. Each experiment was conducted in triplicate to ensure the reliability of the results. The specific optimization steps are outlined as follows, all experimental parameters were systematically optimized using a one-factor-at-a-time approach to ensure clear interpretation of each variable’s individual effect:

- Preliminary screening of crRNA: five designed crRNAs were screened preliminarily.
- CrRNA concentration: the crRNA concentration was adjusted to within the range 20–160 nM.
- Buffer type: Reaction Buffer from EasyZyme BioTech Co., Ltd.(Shenzhen,China) and NEbuffer 2.1 (New England Biolabs, B7202S) were used in these experiments.
- Cas12a protein concentration: the concentration was adjusted to within the range 20–160 nM.
- Reporter concentration: the concentration was adjusted to within the range 50–350 nM.
- Additionally, to shorten the overall reaction time of the detection method, we used the recombinant standard plasmid containing 5.6×10^3^ copies for the following optimizations:
- Adjustment of RAA amplification system timing: The timing of the RAA amplification system was adjusted using the optimized CRISPR/Cas12a reaction system.
- Optimization of CRISPR/Cas12a cutting time: Each of the above experiments was repeated three times.

These optimization measures ensured that the CRISPR/Cas12a reaction system not only effectively generated a fluorescence signal but also allowed for easy visual assessment of the results while minimizing the reaction time required. Through the optimization of the system and timing, the CRISPR/Cas-eqbE-RAA Platform was successfully established.

### 2.5 Specificity of the CRISPR/Cas-eqbE-RAA platform

To assess the specificity of the CRISPR/Cas-eqbE-RAA method for the detection of *S.equi*, nucleic acids from a variety of equine pathogens isolated from different geographical regions were utilized as reaction templates. The pathogens tested included *Streptococcus zooepidemicus*, equine influenza virus (H3 subtype), equine anemia virus equine herpesvirus type 1 and type 4, equine arteritis virus, and *Escherichia coli*. Deionized water (ddH_2_O) was used as the negative control (NC). The specificity was evaluated by measuring the fluorescence intensity using real-time fluorescence quantitative PCR and by visual inspection under UV light.

### 2.6 Sensitivity of the CRISPR/Cas-eqbE-RAA method

To accurately assess the sensitivity of the CRISPR/Cas-eqbE-RAA method, the optimized reaction conditions of RAA-CRISPR/Cas12a-eqbE were utilized. The standard plasmid, starting at a concentration of 5.6 × 10^6^ copies/μL, served as the template. This was serially diluted with deionized water in tenfold increments to create a total of seven gradient concentrations ranging from 5.6 × 10^6^ to 5.6 × 10^0^ copies/μL. These dilutions were used to determine the detection limit of the optimized method.

### 2.7 Evaluation of CRISPR/Cas-eqbE-RAA for clinical sample detection

Nucleic acid from swab samples of 38 clinically suspected equine patients were extracted and simultaneously subjected to both the optimized CRISPR/Cas-eqbE-RAA reaction and the established qPCR method. To ensure the reliability of the results, each experiment was repeated three times. The qPCR setup included: 2× AceQ Universal U + Probe Master Mix V2 (10 μL), *eqbE* primers F/R (10 µM, 1μL), *eqbE*-probe (10 µM, 1 μL), template DNA (1 μL), and ddH_2_O to a total volume of 20 μL. The thermocycling conditions for the qPCR were: an initial denaturation step at 95°C for 3 minutes, followed by 40 cycles of 95°C for 10 seconds (denaturation) and 60°C for 30 seconds (annealing/extension). A Ct value of ≥36 was considered indicative of a negative result, signifying the absence of detectable target DNA in the sample (North et al., 2014).

This comparative evaluation aims to assess the sensitivity and specificity of the CRISPR/Cas-eqbE-RAA method relative to the established qPCR technique, providing insights into the diagnostic accuracy of the CRISPR-based method in clinical settings.

### 2.8 Data analysis

Data processing, graphing, and statistical analyses were conducted using Graphpad Prism 9.5.1 software. Differences between the experimental and control groups were assessed using one-way ANOVA, with Bonferroni corrections applied for multiple comparisons. Statistical significance was determined with unpaired two-tailed t-test and all data were shown as mean ± S.D of 3 replicates. Asterisks indicate **P < 0.01; ***P < 0.001; ****P < 0.0001 and “ns” means non-significant. Images captured under UV light were recorded with an iPhone 14 Pro. The final image processing and layout were performed in the Adobe Illustrator (2024) software.

## 3 Results

### 3.1 Working principle of a CRISPR/Cas12a-eqbE-RAA diagnostic platform for *S.equi* detection

This study aimed to develop a visual diagnostic platform for the detection of *S.equi*, utilizing RAA technology and the CRISPR/Cas12a reaction. To enhance the sensitivity and specificity of the assay while simplifying the operational procedures and minimizing contamination, an integrated reaction strategy was implemented. Specifically, the RAA reaction and the CRISPR/Cas12a reaction were strategically positioned at the bottom and the inner lid of a centrifuge tube, respectively. This design allowed the entire detection process to be conducted without the need to open the centrifuge tube, significantly reducing the risk of contamination.

**Figure 1:**
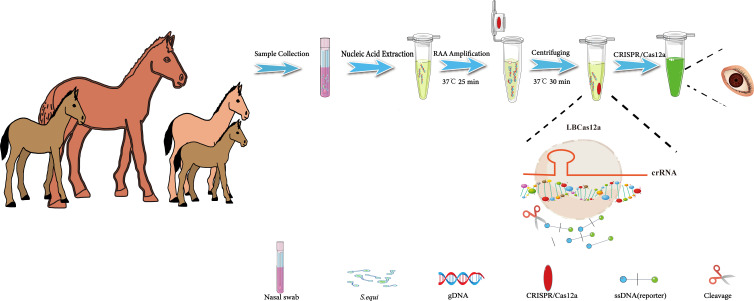
Flowchart of the CRISPR/Cas12a-eqbE-RAA method for the detection of *S. equi*. This flowchart illustrates the developed CRISPR/Cas12a-eqbE-RAA method and its application in the detection of *S. equi*. The procedure includes collection of nasal swabs, extraction of nucleic acids, and conduction of an RAA reaction to amplify the target DNA at 37°C for 25 minutes. The RAA reagents are placed at the bottom of the centrifuge tube, with CRISPR/Cas12a reagents pre-set on the inner wall of the tube cap. Then, the CRISPR/Cas12a reagents are mixed into the tube using centrifugation, and then the CRISPR/Cas12a and RAA products are incubated together at 37°C for 30 minutes to facilitate mixing. Finally, the presence of *S. equi* is visually confirmed by observation under UV light.

The detection process was as follows: Nasal or pharyngeal swabs were collected from horses suspected of being infected with *S.equi*. Nucleic acids were then extracted from these swabs and added to the RAA reaction mixture at the bottom of the tube. Upon completion of the RAA reaction, which exponentially enriched the target sequence, a centrifugation step mixed the CRISPR/Cas12a reaction system with the RAA-enriched target sequence. During this stage, the crRNA specifically recognized and bound to the target sequence, activating the Cas12a protein, which formed a ternary complex and gained the ability to indiscriminately cleave single-stranded DNA. This cleavage resulted in the severing of single-stranded DNA reporter molecules within the system, emitting a bright fluorescent signal. This signal could be visually observed under UV light to confirm the presence of *S.equi* in the sample (**Figure 1**). Additionally, the fluorescence intensity can be quantitatively analyzed using a qPCR detection system to further validate the results.

### 3.2 Establishment of the RAA reaction

**Figure 2:**
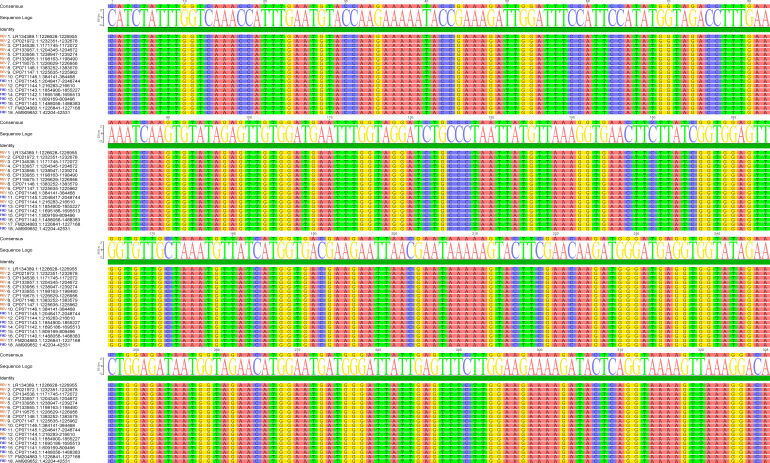
Sequence alignment of the *eqbE* gene segment across various strains of *S.equi*. The left column lists the accession numbers of the sequences analyzed. The alignment demonstrates the high conservation of the eqbE gene segment in this species; the high degree of conservation is critical for the specificity of the CRISPR/Cas12a-eqbE-RAA monitoring system. The sequence logo at the top represents the consensus sequence, highlighting conserved regions.

The success of the CRISPR/Cas12a-eqbE-RAA monitoring system relies on the generation of high-quality target sequences through the RAA reaction. This process is critically dependent on the careful design and selection of primers. We engineered primers to specifically target a highly conserved segment of the *eqbE* gene, which is unique to *Streptococcus equi* subspecies *equi*. Comprehensive sequence analysis, using data from the National Center for Biotechnology Information (NCBI) (https://www.ncbi.nlm.nih.gov/), confirmed the conservation of this segment, ensuring the specificity of our detection method (**Figure 2**).

### 3.3 Optimization and establishment of the CRISPR/Cas12a-eqbE-RAA detection system

**Figure 3:**
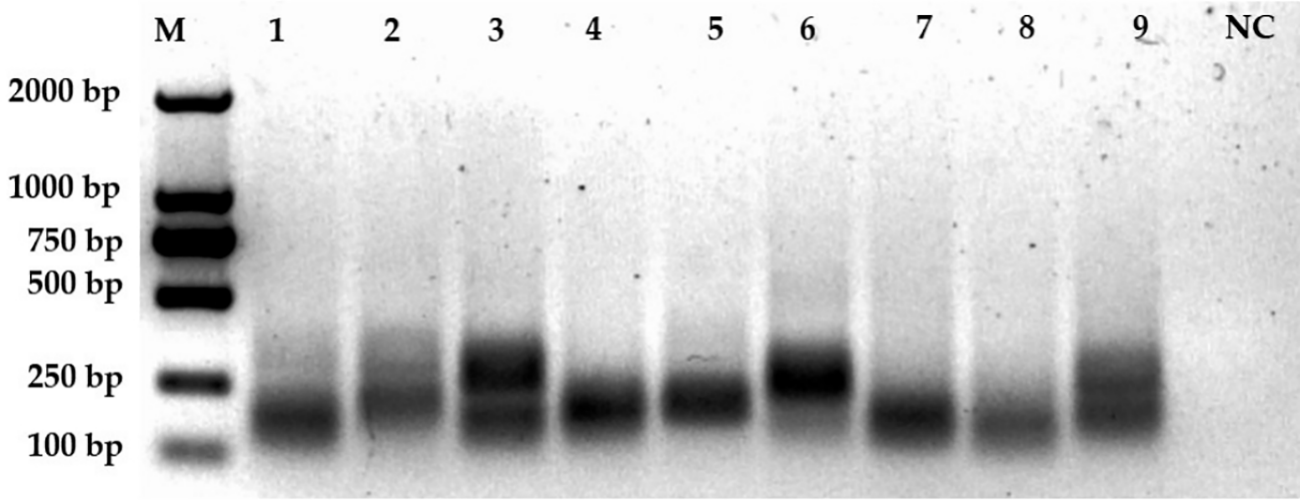
Screening of primer pairs. This figure displays the amplification results of nine primer pairs on an agarose gel. Lane M represents the 2000 bp marker. Lanes 1 to 9 correspond to the amplification results of primer pairs F1R1 (lane 1), F1R2 (2), F1R3 (3), F2R1 (4), F2R2 (5), F2R3 (6), F3R1 (7), F3R2 (8), and F3R3 (9), respectively. The negative control (NC) is shown in the last lane.

To further refine our CRISPR/Cas12a-eqbE-RAA monitoring system, we designed three upstream primers and three downstream primers, resulting in nine potential primer pairs. We tested these pairs using a standard plasmid containing 5.6 × 10^4^ copies to identify the optimal primer pair for amplification. By visual assessment of agarose gels (**Figure 3**) showed that F2R1 (Lane 4) produced the brightest and sharpest target band (300 bp), indicating superior amplification efficiency. In contrast, F2R2 (Lane 5) yielded a visibly fainter target band, while F3R2 (Lane 6) exhibited diffuse smearing characteristic of non-specific amplification. Therefore, F2R1 was selected for optimal performance.

**Figure 4:**
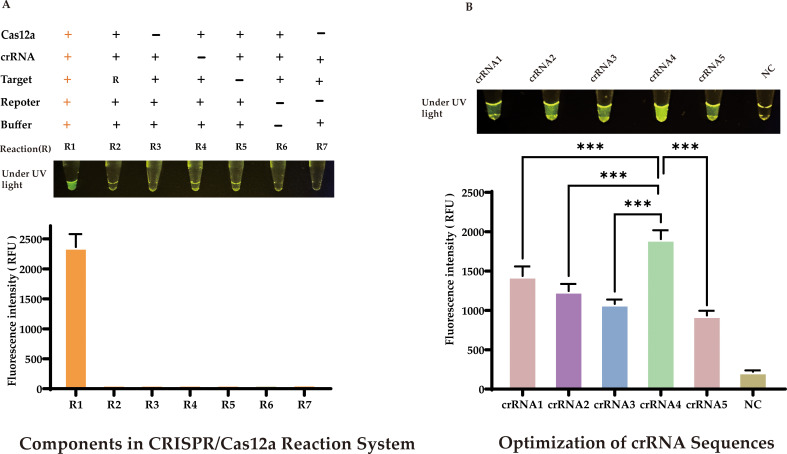
Optimization of CRISPR/Cas12a-eqbE-RAA Detection System **(A)** Component Dependency: Fluorescent signal requires simultaneous presence of target sequence, Cas12a protein, crRNA, and reporter DNA. **(B)** crRNA Screening: Among five crRNAs designed for eqbE gene and PAM preference, crRNA4 yielded maximal fluorescence (visible to naked eye). Statistical notation: ***P < 0.001.

Our approach used the products of RAA amplification in a CRISPR/Cas12a cleavage reaction to enhance cleavage efficiency. We optimized the CRISPR/Cas12a reaction system using 20 ng of the target sequence, and a comprehensive study was conducted to identify the critical components influencing the generation of the fluorescent signal. As depicted in **Figure 4A**, a fluorescent signal was only produced when the target sequence, Cas12a protein, crRNA, and single-stranded DNA (reporter) were all present in the reaction mixture.

CrRNA is crucial in the CRISPR/Cas12a cleavage reaction as it specifically recognizes the target sequence and forms an active complex with Cas12a protein, activating the cleavage reaction. Based on the *eqbE* gene sequence and the PAM site preference of Cas12a, we designed five structured crRNAs (**Table 1**). Screening with a fluorescence quantification system revealed that crRNA4 consistently produced significantly higher fluorescence signals than other crRNAs at various time points and was visually the brightest (**Figure 4B**). Thus, crRNA4 was selected as the optimal crRNA sequence.

**Figure 5:**
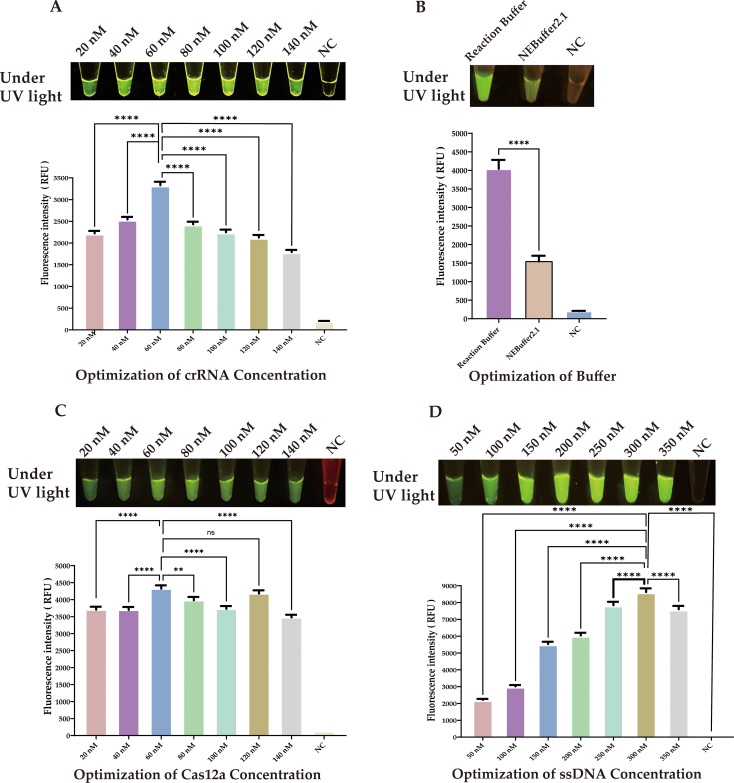
Optimization of CRISPR/Cas12a Components. **(A)** crRNA concentration: 60 nM showed peak fluorescence. **(B)** Buffer system: Reaction buffer outperformed NEBuffer 2.1. **(C)** Cas12a concentration: 60 nM was most effective. **(D)** SsDNA reporter concentration: 300 nM produced strongest signal. Statistical notation: **P < 0.01; ****P < 0.0001; ns: P ≥ 0.05.

We next optimized the concentration of crRNA. Although the differences in fluorescence at various concentrations were subtle to the naked eye, precise quantitative analysis revealed that a concentration of 60 nM crRNA produced a significantly higher fluorescence signal compared to other concentrations (**Figure 5A**, ****P < 0.0001.). Therefore, we chose 60 nM as the optimal concentration for crRNA.

Furthermore, we investigated the impact of different buffers on the efficiency of Cas12a enzyme cleavage. Although NEBuffer 2.1 is commonly used, our experimental data showed that our selected reaction buffer performed better in enhancing cleavage efficiency. (**Figure 5B**, ****P < 0.0001.).

We next determined the optimal concentrations of Cas12a protein and ssDNA through a series of experiments. The results indicated that the optimal concentration for Cas12a protein was 60 nM (**Figure 5C**, ****P < 0.0001.), and for the ssDNA reporter, it was 300 nM, (**Figure 5D**, ****P < 0.0001.).

**Figure 6:**
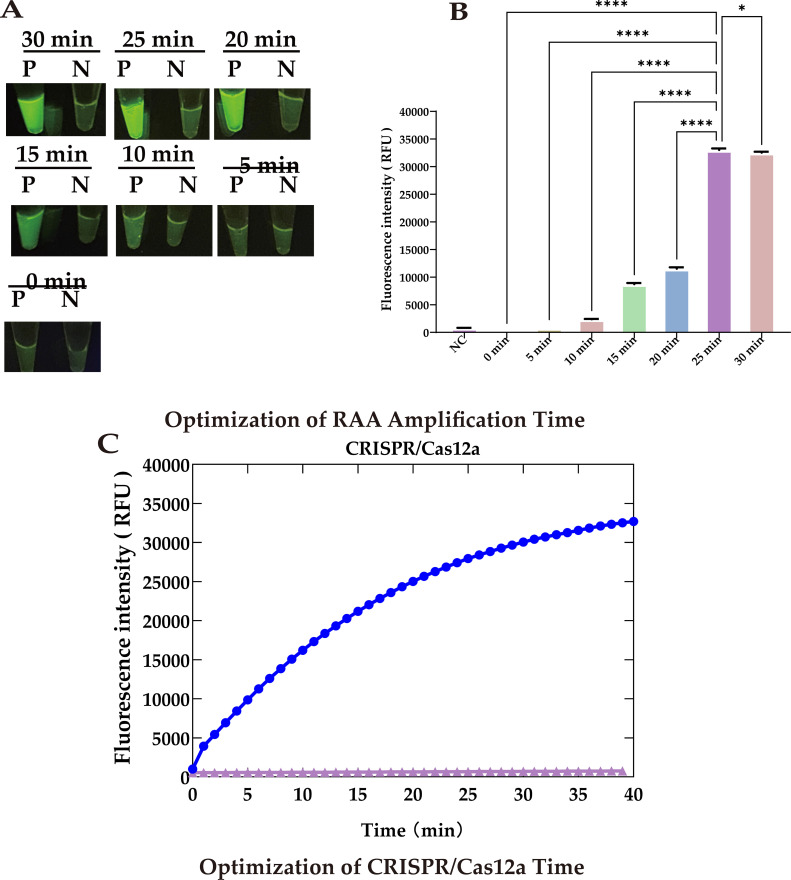
Optimization of reaction time. **(A)** UV images recorded at different RAA amplification times: Peak brightness after 25 minutes. **(B)** Fluorescence at different RAA amplification times: Peaks at 25 minute. **(C)** Optimization of CRISPR/Cas12a cleavage timeThe cleavage rate slows after 30 minutes, defining 30 min as the optimal reaction endpoint. Statistical notation: *P < 0.05; ***P < 0.0001.

To further reduce the total detection time, we utilized 5.6 × 10^3^ copies of template and optimized the reaction times for both RAA amplification and CRISPR cleavage. Our results showed that during RAA amplification, the fluorescence signal increased significantly as the amplification time extended up to 25 minutes (P <0.0001). This increase likely resulted from the accumulation of target DNA, enhancing the activation of the cleavage reaction. However, extending the RAA amplification to 30 minutes led to a decrease (P <0.05) in the fluorescence signal (**Figures 6A, B**).

Similarly, a CRISPR cleavage reaction duration of 30 minutes not only generated a strong fluorescence signal but one that was also clearly observable to the naked eye. Therefore, we set the cleavage time to 30 minutes to shorten the overall reaction time (**Figure 6C**).

### 3.4 Specificity evaluation of the CRISPR/Cas12a-eqbE-RAA detection platform

**Figure 7:**
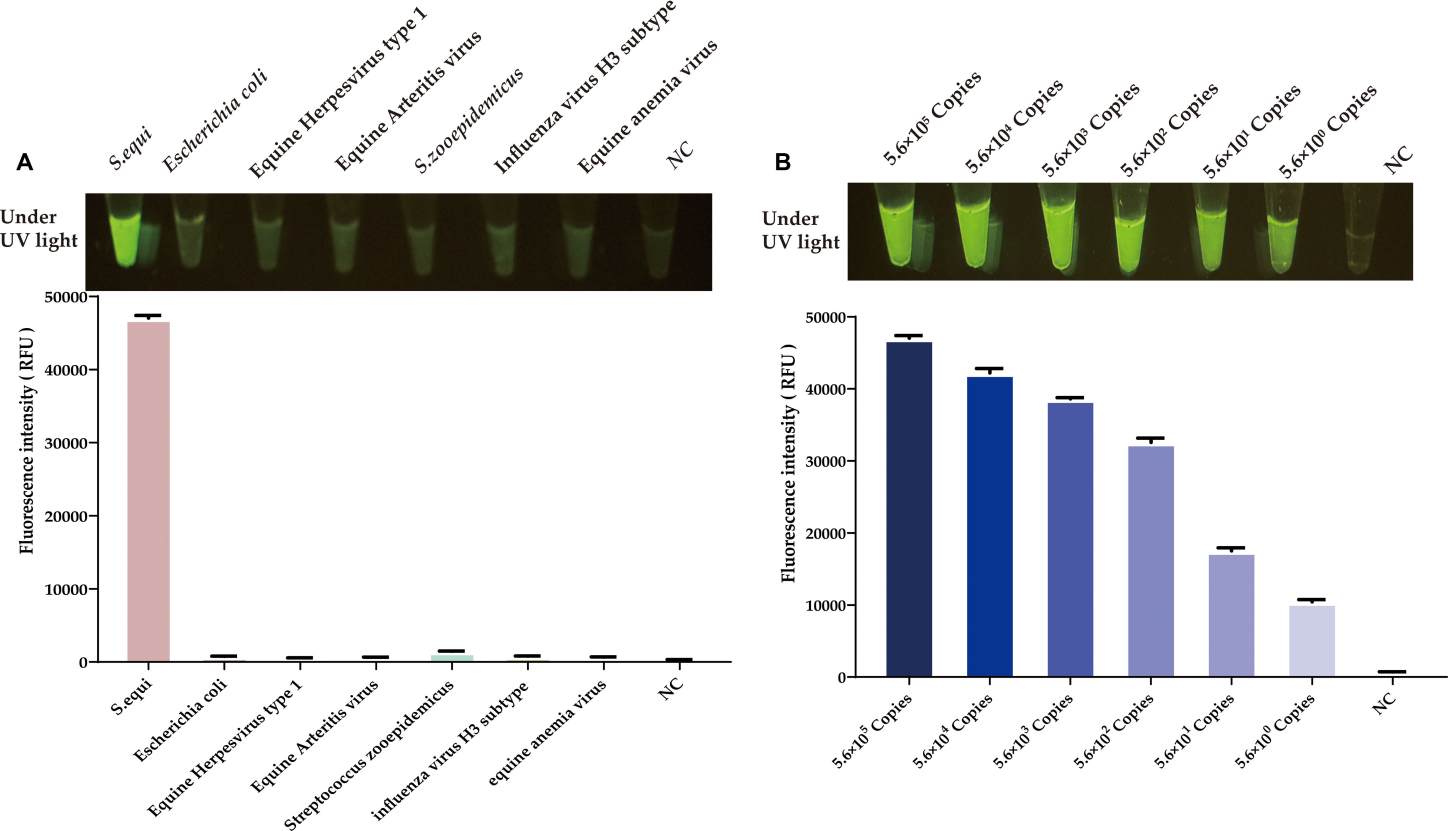
Evaluation of specificity and sensitivity for the CRISPR/Cas12a-eqbE-RAA detection system. **(A)** Specificity evaluation for various equine pathogens and related bacterial species. **(B)** Sensitivity evaluation for tenfold dilutions of the plasmid.

After optimizing the RAA reaction and the CRISPR/Cas12a system, we conducted a specificity assessment on the newly established platform. As depicted in **Figure 7A**, this method precisely identifies the nucleic acids of *S.equi*. Testing against a spectrum of prevalent equine clinical pathogens, including equine influenza virus (H3 subtype), equine anemia virus, equine herpesvirus types 1 and 4, equine arteritis virus, and *Escherichia coli*, as well as the related *S.zooepidemicus*, yielded no significant luminescence or fluorescence signals. This absence of cross-reactivity underscores the high specificity of the CRISPR/Cas12a-eqbE-RAA detection system.

### 3.5 Sensitivity of the CRISPR/Cas12a-eqbE-RAA detection platform

To evaluate the sensitivity of the CRISPR/Cas12a-eqbE-RAA detection system, we performed a series of tenfold serial dilutions on a constructed standard plasmid. The testing results demonstrated that the CRISPR/Cas12a-eqbE-RAA detection platform is capable of detecting as few as 5.6 × 10^0^ copies of the standard plasmid (**Figure 7B**). This indicates that the visualized CRISPR/Cas12a-eqbE-RAA platform exhibits high sensitivity.

### 3.6 Evaluation of the practicality and feasibility of the CRISPR/Cas12a-eqbE-RAA system for testing clinical samples

**Figure 8:**
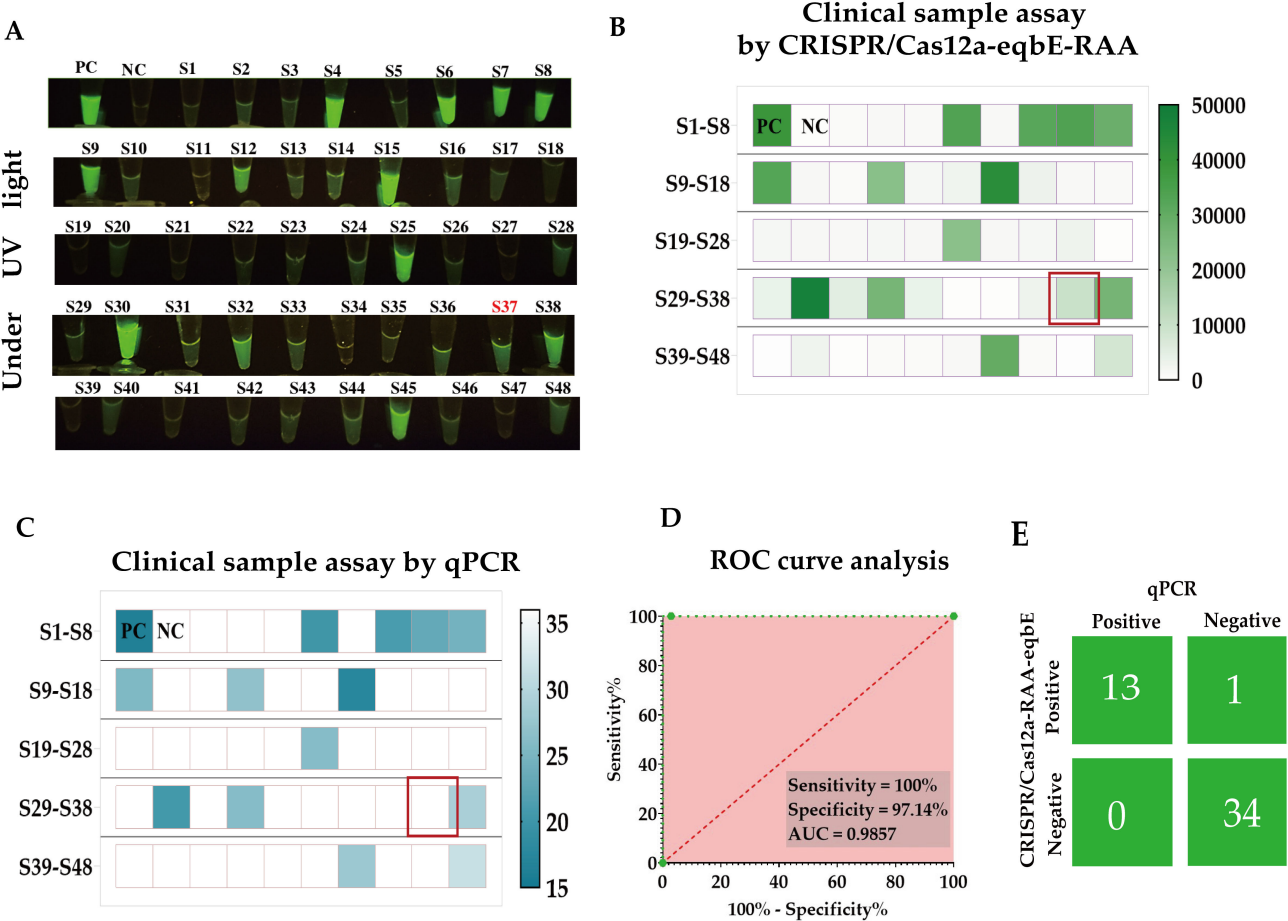
Clinical application of the CRISPR/Cas-eqbE-RAA platform for the detection of *S. equi*. **(A)** Detection of *S. equi* in nasal swabs from clinically suspected equines using the CRISPR/Cas-eqbE-RAA platform, observed with the naked eye under UV light. **(B)** Detection of *S. equi* in nasal swabs from clinically suspected equine patients using the CRISPR/Cas-eqbE-RAA platform. The heatmap displays the fluorescence intensities for each sample, including positive controls (labeled “PC”) and negative controls (labeled “NC”). **(C)** Confirmation of clinical samples using qPCR. The heatmap shows the Ct values for each sample, along with those for positive controls (labeled “PC”) and negative controls (labeled “NC”). **(D)** ROC curve analysis of the detection accuracy of the CRISPR/Cas-eqbE-RAA platform in clinical applications. **(E)** Confusion matrix summarizing the performance of the CRISPR/Cas-eqbE-RAA platform and qPCR assay in discriminating between positive and negative clinical samples.

In this study, we collected nasal swab samples from 48 horses suspected of being infected with *S.equi*, in order to evaluate the performance of the CRISPR/Cas-eqbE-RAA detection platform in practical applications (**Figure 8**). Initially, all samples were screened using the CRISPR/Cas-eqbE-RAA platform. The results indicated that 34 samples tested negative for *S.equi*, while 14 samples were confirmed as being positive (**Figures 8A, B**).

To further validate these findings, we employed real-time quantitative PCR (qPCR) as the gold standard for comparative testing, with a cycle threshold (Ct) of 36 defining positive. The qPCR results were highly consistent with those from the CRISPR/Cas-eqbE-RAA platform, confirming 35 negative samples and 13 positive samples (**Figure 8C**).

Statistical analysis revealed perfect agreement with Cohen’s kappa coefficient (κ) = 0.95 (95% CI: 0.85-1.00) and overall concordance of 97.9% (47/48).Based on these data, we constructed a Receiver Operating Characteristic (ROC) curve (**Figure 8D**) to assess the performance of the CRISPR/Cas-eqbE-RAA platform in detecting *S.equi*. The ROC curve analysis revealed that the platform achieved 97.14% specificit and 100% sensitivity, with an Area Under the Curve (AUC) of 0.9857 (95% CI: 0.9528-1.000; P < 0.0001). This demonstrates the high diagnostic accuracy of the CRISPR/Cas-eqbE-RAA platform.

Overall, the results indicate that the CRISPR/Cas-eqbE-RAA platform has significant potential in the rapid and accurate detection of *S.equi*, and suggesting that it may have many diverse applications in the management and control of equine diseases.

## 4 Discussion

*S.equi* is a major equine respiratory pathogen. It has diverse modes of transmission, including direct contact and indirect environmental spread, and therefore controlling its spread presents a significant challenge. Furthermore, carriers can become silent sources of disease transmission without showing obvious external symptoms, which is a major problem in densely populated equine environments such as racetracks and breeding farms. Therefore, rapid and accurate diagnostic technologies are crucial for the early identification and control of disease spread (Boyle et al., 2016; Durham et al., 2018; Waller, 2014).

The CRISPR/Cas12a-eqbE-RAA system introduced in this study combines the advantages of isothermal amplification and CRISPR/Cas12a technologies to provide an efficient, rapid, and cost-effective detection solution. Crucially, it eliminates dependency on capital-intensive thermal cyclers —unlike compact qPCR systems (e.g., BioRad CFX96 Touch, 12 kg), which remain impractical for field deployment due to power requirements and limited throughput (≤16 samples/run). Our platform achieves laboratory-comparable sensitivity using only portable UV transilluminators or smartphone readers (420–700 USD), enabling deployment in resource-limited equine farms (Huang et al., 2021; Jiang et al., 2022; Jirawannaporn et al., 2022; Sam et al., 2021).

Through single-variable optimization, we systematically calibrated each component of the CRISPR-RAA system to achieve maximum diagnostic efficiency. Initial screening of five crRNA candidates identified the highest-efficiency spacer, while concentration gradients for crRNA, Cas12a, and reporter established optimal stoichiometry. Buffer compatibility was validated across commercial systems (EasyZyme vs. NEB), ensuring reagent robustness. Crucially, RAA amplification peaked at 25 minutes, and CRISPR cleavage optimized at 30 minutes (see **Table 2**). This reaction time can prevents false positives by halting amplification before non-specific products accumulate. The finalized protocol delivers rapid detection (30 min RAA + 15 min CRISPR + 5 min visual readout,50 min total), great sensitivity (1×10^0^ gene copies), and field-ready reliability via closed-tube design—enabling stall-side diagnosis with laboratory-grade accuracy in half the time of qPCR’s lab-dependent process.

**Table 2:** Optimized reaction parameters determined by one-factor-at-a-time (OFAT) analysis.

Parameter	Tested range	Optimal value
Primer Pair	9 variants	F2R1
crRNA	5 sequences	crRNA4
concentration of crRNA	20–100 nM	60 nM
concentration of Cas12a	20–100 nM	60 nM
concentration of Reporter	100–500 nM	300 nM
Reaction Buffer	NEB vs. EasyZyme	EasyZyme
RAA Time	15–35 min	25 min
CRISPR Time	10–40 min	30 min

Although the CRISPR/Cas12a-eqbE-RAA system has shown potential for the rapid detection of *S.equi* in this study, there are some shortcomings that need to be addressed in future research. The single discordant case (CRISPR/Cas12a-eqbE-RAA +/qPCR-) was verified as a false positive by sequencing (**Figure 8E**). This likely originated from amplification product carryover contamination – a recognized challenge in isothermal amplification systems where high-copy amplicons generated at constant temperatures (37°C for RAA) may persist as aerosol contaminants Therefore, similar samples should be considered as potential infections. Additionally, CRISPR/RAA assays exhibit higher per-reaction reagent costs than qPCR (3.60–4.10 USD vs. 1.50–2.40 USD), primarily due to Cas enzyme and proprietary RAA amplification expenses. However this method can fundamentally eliminate dependence on capital-intensive instrumentation. Where traditional qPCR requires 28,000–69,000 USD thermal cyclers and specialized laboratory infrastructure, CRISPR/RAA only necessitates portable UV light sources or smartphone imaging systems costing 420–700 USD, reducing equipment investment by >95%, which enabling sample-to-answer deployment in resource-limited environments such as farms and field surveillance sites. The enhanced portability and operational simplicity facilitate immediate cost recovery in decentralized settings.

## 5 Conclusions

In summary, the CRISPR/Cas12a-eqbE-RAA system offers high sensitivity and specificity. This work establishes a rapid, sensitive, and field-deployable tool for *S. equi* diagnosis. The development of this platform not only provides new technical means for detecting equine infectious diseases but also demonstrates the potential of modern molecular biology in animal disease control. Future efforts should focus on freeze-dried reagent formulations to reduce costs and contamination risks. With further optimization, this system may be widely applied to on-site diagnosis of diverse animal diseases, contributing to global animal health management. With further development and optimization, we hope that this system can be widely applied to the rapid on-site diagnosis of more animal diseases, contributing to global animal disease management and control.

In this study, we developed the CRISPR/Cas12a-eqbE-RAA detection platform for the first time, providing a rapid, sensitive, and cost-effective solution for the detection of the primary equine respiratory pathogen *S.equi*. Integrating the advantages of isothermal amplification and CRISPR technology, this system boasts high sensitivity and specificity. It can effectively detect extremely low pathogen loads and is straightforward to use, making it suitable for on-site rapid application. Particularly applicable in critical settings such as racetracks, the system can accurately differentiate *S.equi* from other pathogens, ensuring equine health and significantly reducing economic losses.

## Data Availability

The original contributions presented in the study are publicly available at: https://doi.org/10.6084/m9.figshare.29928272..
