# Supplementary material for: Integrated CRISPR-Cas12a and RAA one-pot visual strategy for the rapid identification of Streptococcus equi subspecies equi
Source: Front Cell Infect Microbiol. 2025 Aug 21;15:1526516. doi: 10.3389/fcimb.2025.1526516 (PMC12408588; doi:10.3389/fcimb.2025.1526516)
Supplement: Supplementary file 1 [file Table1.docx]

| Name | Sequence (5’- 3’) |
| --- | --- |
| *eqbE*-F1 | CATCTATTTGGTCAAACCATTTGAATGTACCAAG |
| *eqbE*-F2 | CCGAAAGATTGGATTTCCATTCCATATGGTAG |
| *eqbE*-F3 | TGGTAGGATCTGCCCTAATTATGTTAAAGGTG |
| *eqbE*-R1 | CTACCATTATCTCCAGTTCTATACCACCTCATC |
| *eqbE*-R2 | TACCACCTCATCCCATCTTGTTCGAAGTAC |
| *eqbE*-R3 | CCAAGAAACTCAATAATCCCATCATTCCATG |
| *eqbE*-F(qPCR) | ATGTAGCTATGGCAAATGTGGC |
| *eqbE*-R(qPCR) | CAGGTGTTCCTAAGGGTGTT |
| *eqbE*-probe(qPCR) | FAM -AACCTGTTGAAATTAGCCCTTATGATAGTGCG-BHQ1 |
| *Reporter* | FAM-TTATT-BHQ1 |

Supplementary Table 1:primers、probe and reporter sequences
